# Traumatic Injury to the Developing Brain: Emerging Relationship to Early Life Stress

**DOI:** 10.3389/fneur.2021.708800

**Published:** 2021-08-18

**Authors:** Kaila N. Parker, Michael H. Donovan, Kylee Smith, Linda J. Noble-Haeusslein

**Affiliations:** ^1^Department of Neurology, Dell Medical School, University of Texas at Austin, Austin, TX, United States; ^2^Department of Psychology, Behavioral Neuroscience, College of Liberal Arts, University of Texas at Austin, Austin, TX, United States

**Keywords:** early life stress, traumatic brain injury, developing brain, inflammation, immune priming, stress

## Abstract

Despite the high incidence of brain injuries in children, we have yet to fully understand the unique vulnerability of a young brain to an injury and key determinants of long-term recovery. Here we consider how early life stress may influence recovery after an early age brain injury. Studies of early life stress alone reveal persistent structural and functional impairments at adulthood. We consider the interacting pathologies imposed by early life stress and subsequent brain injuries during early brain development as well as at adulthood. This review outlines how early life stress primes the immune cells of the brain and periphery to elicit a heightened response to injury. While the focus of this review is on early age traumatic brain injuries, there is also a consideration of preclinical models of neonatal hypoxia and stroke, as each further speaks to the vulnerability of the brain and reinforces those characteristics that are common across each of these injuries. Lastly, we identify a common mechanistic trend; namely, early life stress worsens outcomes independent of its temporal proximity to a brain injury.

## Introduction

According to the Centers for Disease Control (1), children (age 0–17) are more likely to sustain a traumatic brain injury (TBI), with those 4 years and under at highest risk. Here we focus on the young brain, due to the high prevalence of TBIs in this age group and address how early life stress (ELS) may alter recovery after an early brain injury.

### Evolution of the Injury

TBI results from both a primary insult, due to the direct tearing and shearing of brain structures, and a secondary cascade of adverse events that begins within minutes post injury and includes disruption of the blood-brain barrier, vasogenic and cytotoxic edema, excitotoxicity, neuroinflammation, dysregulation of metabolism, and cell death [see reviews, Simon et al. (2) and Potts et al. (3)]. With low antioxidant reserves, the developing brain is rendered more vulnerable to these adverse secondary events (4–7). Moreover, injury to the developing brain disrupts normal developmental processes, including myelination, synaptogenesis, synaptic pruning, and gliogenesis, each of which contribute to long-term brain function [(8–12) and see review, Semple et al. (13)]. These disruptions and subsequent progressive neurodegeneration adversely affect normal progression of age-dependent behaviors, such as social cognition, social play, social interaction, working memory, and skill acquisition. When these key stages are disrupted during early childhood, risk-taking tendencies, increased social interactions, novelty seeking, emotional instability, and impulsivity may emerge during adolescence (14–19).

## The Developing Brain and TBIs

### Children and TBIs

A child is more vulnerable to a TBI than an adult due to unique physical attributes of the young brain and body. With a larger head to body ratio and weak musculature of the neck (20), the child's brain is more likely to be exposed to greater acceleration/deceleration forces, resulting in a higher incidence of diffuse axonal injury and cerebral edema (21–23). Additionally, the young brain may sustain greater damage from an impact due to a thin calvarium (24, 25). Beyond these general physical features, recovery after an early age TBI is also influenced by characteristics of the lesion, such as severity, location, focal or diffuse patterns of damage, and laterality of injury, each of which may impact outcomes (16, 26–28). Children with large, more diffuse, and/or bilateral injuries show the poorest performance across cognitive domains (15, 17, 18, 26, 29, 30).

Biological sex is also a determinant of recovery after an early age TBI. Beyond genetic and endocrine differences (31), sex differences also manifest in the timing of the closure of sensitive developmental periods, which occurs earlier in males than in females (32). Clinical studies of brain-injured children likewise identify differences between sexes. For example, females who sustain a TBI during childhood are more likely to internalize emotional problems such as depression and anxiety, whereas males may display emotional problems in the form of substance abuse and criminal behaviors (33–36). Similarly, other clinical studies have reported that females have an increased risk for developing emotional and psychiatric disorders after injury, while males present an increased risk for social and behavioral problems (i.e., communication, social cognition, attention/executive function) within the first year following an early age TBI (26, 36, 37).

### Critical Periods of Brain Development

A TBI during the early postnatal period adversely affects maturation of key developmental processes. Brain development spans early gestation to early adulthood (38). During early postnatal development, the brain's acquisition of new functions and capabilities is highly dependent upon experiential and environmental influences (38). Critical periods of brain development are characterized by robust synaptic pruning, myelination, programmed cell death, alterations in density of neurotransmitters, gliogenesis, and white/gray matter differentiation (16, 39–44). While some developmental processes, including the maturation of the immune system and the blood-brain barrier, are mostly complete by birth (45), others, including synaptogenesis, myelination, and programmed cell death, extend well-beyond the postnatal period, and into adulthood (42). In the human brain, synaptogenesis begins before birth and peaks around the age of 3 (40). A subsequent decrease in synaptogenesis coincides with increased synaptic pruning, which continues over the next several decades (42). Programmed cell death peaks during gestation (40) and also extends into adulthood (40). While myelination is most prominent during years 2–3, this process also continues into early adulthood (40, 46). Importantly, each of these developmental processes are critical for normal brain function at adulthood (40).

The first several years of life are considered a sensitive period of growth where key developmental processes shape brain function and behavior at adulthood. The importance of this period of development has been demonstrated in studies of social behavior, sensory experience and cognition. Toddler-aged children are characterized by a high level of activity and sociability (47). Early age brain injuries may alter the shaping and maturation of these behaviors. As sociability continues to develop into adolescence [(48, 49) and see review, Blakemore (50)], a disruption in the toddler aged child may interfere with the proper sequence of age-appropriate social behaviors and increase the risk of psychiatric disorders (51). Children, during this critical period, are also particularly sensitive to sensory experience as it shapes neural circuits involved in basic sensory processes. For example, light and sound shape the formation of the visual and auditory cortices, respectively, and dictate visual and auditory processing (52, 53). Prolonged deprivation of either stimulus during this period results in an impairment in sensory processing later on in life (52–55). Similarly, early age TBI may also result in poorer cognitive outcomes (16, 56–61). This relationship between early age TBI and cognitive abilities is considered non-linear and is likely sensitive to injury at critical periods of plasticity and behavioral development (62, 63). The earlier the age of a TBI, the higher the risk for delayed or arrested development of cognitive and higher-level executive functioning (18, 59, 61).

### Early Life Stress

Children who are exposed to early life stress are at risk for developing long-term psychosocial impairments and chronic illnesses at adulthood (64–69). ELS may encompass a variety of scenarios including extreme poverty, parental loss, malnutrition, domestic/school/community violence, trauma, child neglect, and/or abuse, altered parental behavior (70–80), and institutional rearing (81). ELS impacts many aspects of brain health and development, including metabolism, circadian rhythms, neuroendocrine function, neuro-immune interactions, and oxidative stress (82–87). Children who experience ELS also have a greater risk for diabetes, obesity-related problems, cardiovascular diseases, autoimmune disease, cancer, and depression at adulthood as well as early mortality (64–69, 88, 89).

### The Social Environment and TBI

In a seminal paper, Fletcher et al. (90) questioned why antecedent psychosocial behavior traits, such as adaptive behavior, communication, daily living, and socialization were not considered in studies of brain-injured children. Such questioning has served as a catalyst for subsequent research to examine the moderating role of the social environment before or shortly after an early age TBI. In long term clinical studies of sociocognitive functioning after childhood TBI (18, 19), it was found that, at adulthood, individuals showed poorer emotional perception, as evidenced by deficits in both recognizing and interpreting emotions based upon facial and vocal cues (19). These findings are thought to reflect vulnerability of the immature social brain to this insult, with sociocognitive deficits resulting from disrupted brain development and inability to acquire social skills at the appropriate developmental time (91). Importantly, long term deficits in emotional perception may be linked to a child's socioeconomic status and levels of family intimacy at the time of injury (18). Catroppa et al. (18) reported the first prospective study that compared pre-injury and 6 months post-injury behavioral outcomes with social participation being predicted by both the severity of the TBI and pre-injury deficits, including lower social participation. Subsequent longitudinal studies support these results; children, exposed to a poor social environment prior to a TBI, have greater impairments in psychosocial outcomes, including social cognition and communication compared to brain-injured children with higher socioeconomic status and optimal home environments prior to their injury (14, 91, 92). The results of these early studies indicate that pre-injury demographics such as socioeconomic status and social environment are likely determinants of behavioral recovery after a TBI.

### Pre-clinical Models of Early Age Brain Injuries

Currently, there are two models of TBIs in rodents that have been used to study the consequences of ELS; namely, a focal cortical injury produced by a controlled cortical impactor device, and a more diffuse injury, produced by a fluid percussion device [Table 1, see reviews, Kochanek et al. (103) and Thompson et al. (104)]. Each of these models involves a craniectomy and exposure of the brain. A focal cortical injury is produced by a pneumatically or electronically driven piston that impacts the exposed dura with tightly controlled velocity, depth of penetration and dwell time, producing a consistent injury to proximal cortical and subcortical areas The fluid percussion model is based upon the delivery of a defined pulse of fluid against the intact dura, resulting in brief deformation of the brain (104). Severity of the injury is dependent upon the strength of the pressure wave, which is generated when a pendulum swings from a variable height to strike a plunger in a saline-filled reservoir. This results in delivery of a pulse of saline against the intact dura. Depending upon the severity of the injury, each of these models may result in deficits in learning and memory, social behaviors, hyperactivity, and anxiety- and depression-like behaviors (56, 103, 105–117).

**Table 1:** Pre-clinical models of traumatic injuries to the developing brain.

**Type of injury model**	**Species**	**Sex**	**Description**	**Location of injury**	**Type of injury**	**References**
Controlled cortical impact	Mouse, rat	M	Craniectomy; Impactor tip is set at pre-determined depth and velocity to strike cortical surface	Parietal lobe Frontal lobe	Focal contusion	(14, 74, 76, 93–98)
Fluid percussion injury	Rat, mouse	M, F	Craniectomy; Plastic cork is struck by pendulum dropped from a pre-defined height- saline is delivered to cortical surface	Parietal lobe	Diffuse injury	(74, 93, 98)
Weight drop	Rat, mouse	M, F	Craniectomy; Rod falls from a fixed height to impact cortical surface Closed head; Skull exposed, weighted impactor drops onto intact skull	Parietal lobe	Focal contusion	(74, 98–101)
Impact acceleration	Rat	M	Closed head injury; Rod free-falls from pre-determined height onto exposed skull	Parietal lobe	Diffuse injury	(74, 101, 102)

*While there are 4 commonly used rodent models of TBI to the developing brain (74), only 2 (controlled cortical impact and fluid percussion injury) have been studied following ELS. Male = M; Female+ F*.

## Pre-Clinical Models of ELS

There are two common models of ELS in rodents, the maternal separation model and the limited bedding nestlet model. These models target early brain development that spans birth to postnatal day 21 with notable variations that include the timing and duration of exposure to an impoverished environment and/or maternal separation.

One of the earliest accounts of the maternal separation paradigm used handling or non-handling of rat pups to invoke an early stress (stimulation) response (118). This foundational model examined how neonatal handling affected plasma corticosterone levels and emotionality later on in life (118, 119). The maternal separation model subsequently evolved into the more modern paradigm of physically separating the pups from the mom, resulting in a more pronounced response of the HPA axis (118–126). While maternal separation is suitable for an examination of acute or repeated stressors, the model is not typically applied to chronic stress, which may result in pup exhaustion due to malnutrition and hypothermia (87). Additionally, the maternal separation model may result in inconsistent results and includes many variations of the paradigm (i.e., timing of separation, duration of separation, measure of stress response). The Limited Bedding Nestlet (LBN) model was developed to examine the effects of chronic ELS, in which rodent pups and the nursing dam are exposed to a metal mesh cage bottom and a reduced nestlet square (87). The LBN model produces a robust activation of the HPA axis as a result of erratic and unpredictable maternal care with minimal observer handling (87, 127–132).

### Maternal Separation Model

In this rodent model of childhood neglect (133, 134), the mother is separated from her pups for a defined period of time each day during the postnatal period. The MS model is used by a number of groups (118, 119, 121–126, 135, 136). It results in activation of the hypothalamic-pituitary-adrenal (HPA) axis, as evidenced by elevated corticosterone and altered expression of corticotropin releasing-hormone (CRH) (118, 120, 123, 136, 137). The MS model also results in long-term changes in psychosocial behaviors, including anxiety- and depression-related behaviors. Importantly, there are several variations of this model, including the daily duration of MS (brief vs. prolonged), the timing of the first day of separation, the number of days of separation, if the mother remains in the same room as the pups, and if the pups are maintained on a warming pad while separated from the mother. In some cases, there seems to be habituation to the handling by the observer over an extended period of time (87). In other cases, a brief separation may actually produce positive physiological and behavioral effects later in development, presumably because it replicates the repeated, short periods of separation between mom and pups in the wild, in which the nursing dam leaves her nest to forage for food (135, 138). The desired adverse effects of MS seem to emerge when periods of separation exceed 15 min (139–141). While variation in MS methods may produce some variability in outcomes, there are some key behaviors at adulthood that are common to most, including anxiety- and depression-like behaviors (51, 142–145). Moreover, these models typically show an exaggerated response of the HPA axis, a hallmark of ELS, immediately after the separation period that extends well into adulthood (51, 146–150).

### Limited Bedding Nestlet Model

In the LBN model, the mother rears her pups on an altered cage bottom, typically metal mesh, with a reduced amount of a nesting material during the first week of postnatal life. This model creates a stressful environment, resulting in altered maternal behavior toward her pups (neglect, abuse, and hypervigilance) (87, 89, 127, 129, 131, 132, 151–159) and an exaggerated response by the HPA axis of the pups, based on changes in CRH and elevated corticosterone levels, that extends into adulthood (87, 129, 130, 155, 160). This paradigm, usually applied from P2-P9, produces long-term behavioral impairments such as anxiety, fear learning, depression, anxiety, reduced sociality (play behavior), and deficits in spatial learning and memory later in life (87, 89, 129, 131, 132, 151, 153–155, 157–159). A key strength of this model is that there is opportunity to continuously monitor maternal care and interaction with her pups without any confounding effects, resulting from handling by the experimenter.

There is reduced pup weight during and after the period of LBN (129, 130, 161, 162), which in some cases persists into adulthood (132). Although the LBN model shows variability in body development, it consistently results in altered metabolism, as evidenced by changes in brown adipose tissue and in circulating leptin and glucose levels. The lasting metabolic effects of LBN may be a result of the combination of the quality and quantity of nutrition, stress hormones, and sensory stimuli from the mother (163).

## ELS and Immune Priming

While the immune response to a TBI contributes to secondary damage (113, 164–167), we have yet to fully understand the interaction between ELS and TBI in this context. ELS may prime the immune system, leaving it sensitized to inflammatory reactions later in life.

### Causes and Effects of Immune Priming

Exposure to a wide variety of early-life insults may elicit a persistent immune-sensitized condition in the brain, such that a subsequent insult produces a heightened inflammatory response. This phenomenon is referred to as “immune priming.” Early life insults that have been shown to cause immune priming include infections (168, 169), seizures (170), early postnatal alcohol exposure (171), *in utero* stress (172), and as discussed in detail below, ELS (99, 173–175). Insults in the early period of life may produce life-long sensitization, creating immune cells that remain primed for many months in rodents and decades in humans (99, 176). Immune priming typically involves circulating immune cells, peripheral macrophages, astrocytes, or even neurons, but the most heavily implicated cells in immune priming of the CNS are the brain's resident immune cells, microglia, which undergo a phenotypic shift, producing much faster and more robust responses to subsequent immune signals (177–179).

### The HPA Axis and Inflammation

In response to a stressor, the body activates the HPA axis. The hypothalamus, initially stimulated by the sympathetic nervous system, releases corticotropin-releasing hormone into the nearby pituitary gland, which in turn releases adrenocorticotropic hormone (ACTH) into the blood stream. Upon reaching the adrenal glands, ACTH causes release of glucocorticoids (GC), namely corticosterone in rodents and cortisol in humans. GCs then act on cells expressing glucocorticoid receptors throughout the body including the brain. In this way the stress signal is amplified and extended to enable a whole-animal response in the minutes and hours following a stressor. In general, GCs have an anti-inflammatory effect, inhibiting lymphocyte proliferation, reducing expression of pro-inflammatory cytokines and inhibiting production of anti-inflammatory cytokines (93, 180–183). This is especially true when GC levels are high, since, of the two GC receptors, the one that predominates in response to elevated levels of GC has a distinctly more anti-inflammatory signaling profile (94, 95). How then, does ELS lead to chronic inflammation and immune priming? One part of the puzzle may be that GCs elicit responses in the brain that are quite different than the primarily anti-inflammatory effect in the periphery. In addition to microglia, neurons and astrocytes in the brain also express GC receptors and elevated GCs can weaken these cells, compromising their ability to withstand further insult (96–98, 100, 101). Frank et al. have recently demonstrated that either stress or exogenous GCs produces immune-primed hippocampal microglia that, when challenged with lipopolysaccharide (LPS) *ex vivo*, secrete increased proinflammatory cytokines (102, 184). Furthermore, this effect is long-lasting, with these microglia still exhibiting a primed phenotype 28 days after a single stressor. One intriguing potential mechanism for GC-mediated priming of microglia is the nod-like receptor protein 3 (NLRP3) inflammasome. This protein complex is induced by GCs, is capable of regulating proinflammatory cytokine release, and has been implicated in microglial immune priming (102, 184–187).

### The HPA Axis and TBI

TBI results in a suppression of the HPA axis [see review, Tapp et al. (188)]. As described above, the HPA axis responds to stressors by releasing corticotropin-releasing hormone (CRH) to the pituitary gland, which releases ACTH into the bloodstream. ACTH causes a release in glucocorticoids, like corticosterone (CORT). Under normal conditions, HPA axis activity is regulated by glucocorticoid receptors (GR) in the hypothalamus, pituitary, and adrenal glands. In addition to damage to subcortical areas (189), TBI causes a release of CORT in the brain. GR involved in the HPA axis negative feedback loop also become damaged from TBI, resulting in an excess of CORT. The pituitary is particularly vulnerable to injury-induced dysfunction, which results in a decreased release of ACTH and cannot stimulate the adrenal glands. The lack of stimulation results in decreased CORT release from the adrenal glands, resulting in an aberrant altered stress response. Experimental models of TBI have examined HPA axis suppression in rats, in which CORT was diminished in injured mice at 7 and 21 days after injury (190, 191). Excessive glucocorticoid release and a suppressed HPA axis response after TBI causes microglial priming and increases inflammatory cytokine expression, resulting in neuronal death (192, 193). This maladaptive chronic inflammatory response contributes to the development or worsening of psychiatric disorders later in life, such as depression (194, 195). The aberrant interaction between the persistent neuroendocrine response and compromised psychiatric behavior illustrates the importance of HPA axis dysfunction and long-term TBI recovery.

### ELS Animal Models and Immune Priming

To date, only a handful of studies have examined immune priming or markers of chronic inflammation in the context of either the MS or LBN model of ELS (Table 2). Most of these have reported robust and long-lasting effects of ELS on cytokine expression. Reus et al. used an MS model in rats (P1-P10, 3 h/day), and quantified multiple cytokines at P20, P30, P40, and P60 in 3 different brain regions (99). They found persistently increased levels of the proinflammatory cytokines IL-1β, IL-6, and TNFα, as well as decreased levels of anti-inflammatory cytokine IL-10 (see Table 2 for details). Wang et al. employed a rat MS model (P2-P20, 4 h/day) and reported elevated pro-inflammatory IL-1β, IL-6, and TNFα protein in the hippocampus and elevated TNFα protein in the prefrontal cortex at P60 (173). Three studies, all from the same group and using an MS model in mice (P2-P14), reported similar results at between P50 and P60; that is, elevated hippocampal mRNA for pro-inflammatory cytokines IL-1β and TNFα, as well as for the inflammasome protein NLRP3 (196–198). Saavedra et al., using a rat MS model (P1-P14, 3 h/day) did not examine cytokines but found an increased proportion of hippocampal microglia that maintained an activated phenotype when examined long after ELS, at between P140 and P170 (174). Sagae et al. utilizing an LBN model (P3-P9) in rats, also reported elevation in circulating pro-inflammatory cytokines TNFα and IL-6 at P98 (175).

**Table 2:** Pro-inflammatory cytokines after ELA in rodents.

**ELS model**	**Cytokines**	**Time of cytokine measurement**	**Findings**	**References**
Maternal separation (P4-11)	•IL-1β •IL-6 •TNFα	P20, P30, P40, P60	**IL-1β** •P20: ↑HPC, no change in serum or PFC •P30: ↑HPC, PFC, Serum •P40: ↑HPC, no change in serum or PFC •P60: ↓HPC, ↑Serum, no change in PFC **IL-6** •P20: ↑HPC, no change in serum or PFC •P30: ↑HPC, no change in serum or PFC •P40: ↑HPC, Serum, PFC •P60: ↑HPC, PFC, no change in serum **TNFα** •All time points: ↑HPC, Serum, PFC	(142)
Maternal separation (P2-20)	•IL-1β •IL-6 •TNFα	P65	•IL-1β: ↑HPC, no change in PFC •IL-6: ↑HPC, no change in PFC •TNFα: ↑HPC, ↑PFC	(143)
Maternal separation (P2-14)	•IL-1β •TNFα	P50	•IL-1β: ↑HPC mRNA •TNFα: no change HPC mRNA	(158)
Maternal separation (P2-14)	•IL-1α •TNFα	P60	•IL-1β: ↑HPC mRNA •TNFα: ↑HPC mRNA	(160)
Maternal separation (P2-14)	•IL-1β •TNFα	P60	•IL-1β: ↑HPC mRNA •TNFα: ↑HPC mRNA	(159)
Maternal separation (P1-21)	•C-Reactive protein •IL-6	P21, P28	•**C-Reactive Protein** •P21 + 28: ↑Plasma **IL-6** P28: ↑HPC mRNA	(162)
Limited bedding nestlet (P2-9)	•IL-6 •TNFα	P98	•IL-6: ↑Serum •TNFα: ↑Serum	(145)
Limited bedding nestlet (P2-9)	•IL-1β •IL-6 •TNFα	P9, 4mo, 10mo	**IL-1β** •P9: ↑HPC mRNA •4 mo: no change •10 mo: inflammation resolved **IL-6** •P9: no change •4 mo: ↓HPC mRNA •10 mo: inflammation resolved **TNFα** •All time points: No change	(161)

*HPC, Hippocampus; P, Postnatal day; PFC, Prefrontal Cortex; P, Postnatal day; IL-1β, Interleukin-1β; IL-6, Interleukin-6; TNα, Tumor necrosis factor alpha*.

Other studies have found smaller or more subtle impacts of ELS models on cytokines. Hoejimakers et al. used a LBN model from P2-P9 in mice and reported increased hippocampal expression of IL-1β at P9, immediately after stress, but decreased hippocampal IL-6 mRNA at 4 months and no differences in any pro-inflammatory cytokines at 10 months (199). Additionally, these investigators reported an increase in CD68 immunoreactivity, characteristic of activated microglia, at 4 months after stress, but not at 10 months. Delpech et al. (200) used a brief MS model (P1-P21, 15 min/day) in mice, following ELS at P21 and at P28 and demonstrated an elevation of serum c-reactive protein, a marker of immune activation. At P28 however, there was no effect of ELS on the number and morphology of hippocampal microglia, that had been seen at P21. They also reported elevated IL-6 mRNA from microglia isolated from the hippocampus at P28.

Perhaps the variability of results from ELS models is not surprising given the differences both in the details of the stress paradigms and in the methodology employed to measure cytokines and other features of immune priming. In explaining the differences between the MS studies (99, 173, 174, 196–198), it seems that the duration of the separation may underly the stark differences in results between Delpech et al. (200) (15 min/day) and the rest (3–4 h/day). In the case of the two LBN studies (175, 199), differences may arise from the quite disparate means of cytokine quantification [protein in serum for Sagae et al. (175) vs. hippocampal mRNA for Hoeijmakers et al. (199)]. There may also be species differences in how the immune systems of mice and rats respond to ELS, as several of the studies that found the most robust signs of immune priming were in rats (99, 173–175), while the two with the weakest evidence of immune priming were both evident in mice (199, 200).

### Immune Priming by ELS in Humans

In humans, childhood adversity has been linked to a chronic inflammatory state (201–204), as well as to diseases associated with inflammation, such as cancer, cardiovascular disease, diabetes, and arthritis (64–69, 88, 89). Many studies have examined the relationship between socioeconomic status during childhood and inflammation, typically measured by plasma c-reactive protein (205). Such studies may be complicated by controlling for covariates, such as adult socioeconomic status. A recent meta-analysis examined 35 such studies and found a significant relationship between childhood socioeconomic status and the profile of adult inflammation, but this relationship did not survive when adjusted to factor out adult socioeconomic status (205). Ehrlich et al. (201) examined whether teens' early-life adversity scores, generated from interviews, were associated with differences in their inflammatory profiles. Rather than rely on cytokine or c-reactive protein expression, inflammation was quantified by *ex vivo* challenge of monocytes, obtained from blood samples, with either lipopolysaccharide alone or with lipopolysaccharide in combination with varying concentrations of GC. IL-6 secreted into the culture media was quantified, and a cluster analysis was performed. ELS was associated with higher inflammation clusters, indicating persistent immune priming by early life adversity in this population.

## ELS and Brain Injury

Despite the clinical relevance, there are few preclinical studies that have examined brain injuries after exposure to LBN, brief maternal stress (BMS) or prolonged maternal stress (PMS) [Table 3, (133, 134, 206–210)]. Thus, there is substantial opportunity to build upon what has been reported, focusing on the unanswered questions, with the end goal of optimizing recovery in brain-injured children who have experienced prior ELS.

**Table 3:** ELS prior to neonatal hypoxia-ischemia, stroke or TBI.

**Species and sex**	**Ages at separation and duration**	**Injury type**	**Age at injury**	**Timing and outcomes**	**ELS + injury behavioral findings**	**Findings**	**References**
Mouse/F	P1-14: 15 min/day BMS	Stroke	P100-110	•24, 72 h, 7 d: Behavior CBF •24 or 72 h: CORT •48 h: Edema, Histology •12 h: RT-PCR	•Locomotion: No change •Paw Preference: ↓Contralateral paw	•Histology: ↑infarct volume •RT-PCR: ↑IL-1B ↑TNFα •CBF: No Change •CORT: ↓intra-ischemia •Edema: ↑Edema	(158)
Rat/M+F	P3-7: 30 min/day BMS OR 8 h/day PMS	HI	P7 or P135	•P7-Adult: CORT •P13+P120: Histology •P10+P120: Physiologic measures	Not measured	•CORT: ↑P7, P9-17, P135 •Histology: ↑Atrophy •Injury Scale: Worsened score •Physiology: ↑Hyperglycemia	(159)
Rat/M+F	P1-6: 180 min/day PMS OR 15 min/day BMS	HI	P7	Adult: Behavior, Histology	•↓Spatial acquisition memory •No change in object recognition •↓No change in motor behavior	•Infarct Size: No change •CC: No change	(160)
Rat/M+F	P1-6: 180 min/day PMS OR 15 min/day BMS	HI	P7	Adult: Behavior, Histology	•↑Anxiety •↑Spontaneous movement •No change depression	•Histology: ↓Synaptophysin in HPC ↓BDNF in HPC	(161)
Rat/M	P2-14: 180 min/day PMS	TBI (Mild)	Adult	Adulthood: Behavior, Histology, CORT	•↓Memory retention •↓Spatial working memory	•Histology: ↑Cortical atrophy, ↑HPC atrophy •CORT: ↑Level	(87)
Mouse/M	P1-21: 180 min/day PMS	TBI (Mild)	P21	Adolescence: Behavior, Histology	↓Spatial learning + memory	Histology: ↓Cell proliferation, ↑Iba-1	(88)
Mouse/M	P1-21: 180 min/day PMS	TBI (Mild)	P21	Adolescence: Behavior, ELISA, RT-PCR	No Change in executive functioning	•ELISA: ↑IL-1β, ↑CORT •RT-PCR: ↑CRH, No Change AVP	(162)

*AVP, Antidiuretic Hormone; P, Postnatal day; CC, Corpus Callosum; HPC, Hippocampus; BDNF, Brain-derived neurotrophic factor; CBF, Cerebral blood flow; CORT, Corticosterone; HI, Hypoxia Ischemia; M, Males, F+, Females; TBI, Traumatic Brain Injury; BMS, brief maternal separation; PMS, prolonged maternal separation*.

### ELS + Stroke

Although risk of stroke increases with age, incidence of stroke may occur at any age, including children (211). To date there is only one preclinical study that has examined the relationship between ELS and stroke [Table 3, (206)]. In this study, mothers were briefly separated (BMS) from their pups on a daily basis from P1-P14, a sensitive period of brain development. After reaching adulthood, animals were subjected to an occlusion of the middle cerebral artery followed by reperfusion. There were several findings that distinguished BMS in combination with stroke from controls. These animals showed a pronounced elevation of proinflammatory cytokines IL-1β and TNFα, vasogenic edema, and higher mortality compared to BMS alone. Such findings build upon other studies showing enhanced expression of pro-inflammatory cytokines IL-1β, TNFα, and IL-6 as a result of ELS exposure (99, 175, 199, 200). BMS in combination with stroke also resulted in an impairment of sensorimotor function compared to controls, based upon paw preference using the cylinder test (212, 213). It is noteworthy that there were no changes in corticosterone, either pre- or post-injury compared to relevant controls. While others have reported elevated levels of corticosterone at adulthood after BMS alone (87, 99, 175, 199, 200), the duration of maternal separation may, at least in part, account for these differences. In this stroke study, the duration of BMS was 15 min/day over a period of 2 weeks. In contrast, those studies that detected elevated levels of corticosterone at adulthood after BMS alone (146–150, 214–221), reported a duration of 180 min/day or longer. Collectively, these findings provide the first evidence that ELS in combination with stroke at adulthood elicits a pronounced immune response and adversely affects post-stroke sensorimotor recovery.

### ELS + Perinatal Brain Injury

Neonatal hypoxia ischemia (HI), the most common form of perinatal brain injury, results in neonatal encephalopathy and long-term disabilities (222).

Several preclinical studies have examined the consequences of ELS in combination with HI [Table 3, (207–209)]. Early studies evaluated ELS using BMS (15 min/day) or PMS (180 min/day) exposure to MS on P3-P7, followed immediately by HI, and then studied shortly after HI or at adulthood (207). Prior exposure to PMS and neonatal HI resulted in elevated levels of corticosterone shortly after the time of injury. Histological findings, based upon pathological scoring of hematoxylin stained sections, suggested enhanced damage to white matter in the thalamus and internal capsule. Studies of HI at adulthood showed altered metabolism, as evidenced by elevated levels of glucose and insulin compared to BMS or PMS alone.

A later study focused on the long-term consequences of BMS or PMS in combination with HI on hippocampal functioning at adulthood [Table 3, (208)]. After BMS or PMS from P1-P6, animals were exposed to HI shortly thereafter and then were evaluated at adulthood. While ELS in combination with HI showed no differences in non-spatial recognition (novel object recognition and novel placement test), there were impairments in spatial learning and memory, as measured by the Morris Water Maze, compared to either insult alone.

Lastly, a follow up study focused on the interaction of ELS and HI in the context of synaptic integrity in the hippocampus and metrics of emotionality [Table 3, (209)]. Animals were exposed to PMS and subsequent HI and thereafter evaluated at adulthood for anxiety- and depressive-like behaviors, based upon performance in the elevated plus maze and the forced swim test, respectively. While HI followed by PMS resulted in a more pronounced anxiety-like phenotype, compared to either insult alone, there was no evidence of depressive-like behavior across any groups. Subsequent histological analyses of the dentate gyrus revealed altered long-term synaptic plasticity as evidenced by a reduction in levels of brain-derived neurotrophic factor and synaptophysin in the hippocampus compared to either PMS or HI alone. These results indicate that cell survival and synaptic density in the hippocampus are particularly vulnerable to the additive effect of MS and HI (209).

### ELS + TBI

ELS has been evaluated in pre-clinical models of TBI with variables that include the type of injury (focal vs. diffuse), the age at time of injury, and the timing of outcomes. Sanchez et al. (133) (Table 3), examined how prolonged ELS influences hippocampal-related function after a TBI at adulthood. Animals were exposed to daily PMS (180 min/day) from P2-P14 followed by a mild fluid percussion injury at adulthood. Behavioral assessments were conducted 2, 3, and 4 weeks after injury. Based on contextual fear learning (2 weeks post injury), brain-injured animals, reared in PMS, showed less freezing after the cue compared to controls. Animals were subsequently tested using the Morris Water Maze at 3–4 weeks post injury. The group with PMS in combination with TBI showed deficits in spatial learning as well as greater cortical and hippocampal atrophy compared to other conditions. At 8 weeks post injury, corticosterone levels were highest in PMS in combination with TBI.

An alternative approach examined how PMS (P1-P21) is influenced by a mild TBI at P21 [Table 3, (134)]. In these experiments, a mild focal injury was produced by a controlled cortical impact. Deficits in spatial learning and memory were most pronounced in brain-injured adolescent rodents exposed to both PMS and TBI. Although there was no difference in the lesion volumes across all groups, PMS prior to TBI resulted in an increase in activated microglia and a reduction in proliferation of the markers bromodexoyuridine and the nuclear protein Ki67 in the hippocampus. Taken together, these findings suggest that PMS prior to an early age mild TBI, results in more profound activation of microglia, which, in turn, adversely affects neurogenesis and hippocampal-dependent behaviors (223).

A follow-up study, using the same model of PMS and TBI, examined cognitive flexibility and thereafter measured pro-inflammatory cytokines, IL-1β, TNFα, and IL-6 in the prefrontal cortex and hippocampus. Cognitive flexibility was measured using the attentional shift task in early adolescence, whereby mice learned how to discriminate between positive odors and associate this experience with a cue (210, 224, 225). Mild injury had a significant impact on the first reversal of the attentional shift task. However, this was not worsened by prior exposure to MS. IL-1β, elevated in the hippocampus, was highest in those animals exposed to both PMS and TBI compared to controls. These findings suggest that PMS in combination with a mild TBI results in a heightened inflammatory response compared to either condition alone. Although there was no additive effect seen on cognitive flexibility or in IL-1β in the prefrontal cortex, the authors suggest that IL-1β may be involved in crosstalk between hippocampal and cortical-related cognitive impairments seen after an early age mild TBI.

## Where Do We go From Here?

There are a number of research opportunities that could build upon our current knowledge of the interactions between ELS and recovery after a brain injury. Here we address several basic directions.

### Consider Alternative Models of ELS

ELS has profound adverse effects on brain development and results in both physical and psychological sequelae at adulthood. Few preclinical studies have addressed how ELS may influence recovery after brain injury. And, of these studies, ELS has only been studied in the context of MS (133, 134, 206–210). As ELS represents a broad spectrum of adverse conditions including physical, sexual and emotional forms of abuse and neglect (226), there is a need to address alternative models of ELS, including LBN, as well as others that capture a broader range of adverse exposures.

### Injury Severity as a Modifier of Recovery After ELS

Two of the most commonly used rodent models of TBI, controlled cortical impact and fluid percussion injury, have been studied in the context of ELS (133, 134, 210). The severity of the injury likely influences recovery after ELS. This raises the possibility that very mild forms of TBIs, such as concussions, which present with nominal changes at structural and behavioral levels, may, in fact, be sensitive to prior ELS and, as such, result in broader pathological and behavioral findings. Understanding the relationships between ELS and mild TBIs has broad implications, including how we manage concussions in youth sports.

### Sex as a Biological Variable

There are few studies of ELS in combination with TBI that include both males and females in the experimental design (Table 3). There is evidence that speaks to the complexities of TBIs, where variables such as the severity and type of insult may be differential modifiers between sexes. Thus, from simply the perspective of TBI alone, sex as a biological variable should be a key element in the experimental design [see review, Gupte et al. (227)]. Importantly, in a scoping review of both clinical and preclinical studies, Gupte et al. (227) have indicated that variables such as injury severity and nature of the injury interact differently based upon sex and that these differences influence long-term outcomes.

### Genetics and Epigenetics

Genetics, including both gene variants and epigenetics, play a central role in how a brain recovers after ELS [see review, Fogelman and Canli (226)]. Similarly, genetics, and in particular epigenetics, also contribute to heterogeneity in recovery after a TBI, as evidenced in both preclinical models and in human studies [see reviews, Bennett et al. (228), Cortes and Pera (229), Treble-Barna et al. (230), and Kurowski et al. (231)].

### Immune Function

ELS results in persistent immune priming (201) [and see reviews, Neher and Cunningham (232), and Fagundes et al. (233), von Leden et al. (234)]. We have yet to address how this priming may alter the immune response after a TBI. We and others have reported that the developing brain is sensitive to early cytokine exposure and, in fact, an early age TBI results in an enhanced immune response that is, in part, related to the prolonged recruitment of leukocytes to the injured brain (234). Thus, these collective findings support a further investigation into inflammatory responses, mediated by ELS, that may be magnified after a subsequent TBI.

### ELS, TBI, and Plasticity

There are varying thoughts regarding plasticity after an early age lesion [see review, Giza and Prins (235)]. One viewpoint is that a younger brain has the ability to undergo significant reorganization and recovery after an injury and that ongoing brain development may support recovery processes. This is in contrast to others who consider the vulnerability of the young brain, where growth and formation of circuitry may be compromised by injury during critical periods of brain development. To address these differing viewpoints, further studies are needed to address factors that may influence outcomes, including age at time of injury in the context of brain development, severity and location of the injury, and the type of injury (focal and/or diffuse), as well as a broader viewpoint on plasticity that takes into account both its beneficial and adverse consequences.

## Author Contributions

KP, MD, and LN-H contributed equally to writing and editing of this manuscript. KS contributed to formatting of tables, interpretation of findings, and fact checking of references. All authors have contributed to read and approved the final version of the manuscript.

## Conflict of Interest

The authors declare that the research was conducted in the absence of any commercial or financial relationships that could be construed as a potential conflict of interest.

## Publisher's Note

All claims expressed in this article are solely those of the authors and do not necessarily represent those of their affiliated organizations, or those of the publisher, the editors and the reviewers. Any product that may be evaluated in this article, or claim that may be made by its manufacturer, is not guaranteed or endorsed by the publisher.
